# Whole-Body Imaging to Assess Cell-Based Immunotherapy: Preclinical Studies with an Update on Clinical Translation

**DOI:** 10.1007/s11307-021-01669-y

**Published:** 2021-11-23

**Authors:** Noriko Sato, Peter L. Choyke

**Affiliations:** 1 Molecular Imaging Branch, Center for Cancer Research, National Cancer Institute, National Institutes of Health, Bldg. 10/Rm. B3B406, 10 Center Dr, Bethesda, MD 20892 USA; 2 Molecular Imaging Branch, Center for Cancer Research, National Cancer Institute, National Institutes of Health, Bldg. 10/Rm. B3B69F, 10 Center Dr, Bethesda, MD 20892 USA

**Keywords:** Imaging, Cell-based immunotherapy, Positron emission tomography, Magnetic resonance imaging, Reporter gene imaging, Immune cell, Zirconium-89 oxine, Fluorine**-**19 perfluorocarbon, Superparamagnetic iron oxide nanoparticles

## Abstract

In the past decades, immunotherapies against cancers made impressive progress. Immunotherapy includes a broad range of interventions that can be separated into two major groups: cell-based immunotherapies, such as adoptive T cell therapies and stem cell therapies, and immunomodulatory molecular therapies such as checkpoint inhibitors and cytokine therapies. Genetic engineering techniques that transduce T cells with a cancer-antigen-specific T cell receptor or chimeric antigen receptor have expanded to other cell types, and further modulation of the cells to enhance cancer targeting properties has been explored. Because cell-based immunotherapies rely on cells migrating to target organs or tissues, there is a growing interest in imaging technologies that non-invasively monitor transferred cells *in vivo*. Here, we review whole-body imaging methods to assess cell-based immunotherapy using a variety of examples. Following a review of preclinically used cell tracking technologies, we consider the status of their clinical translation.

## Introduction

Immunotherapy aims to stimulate or suppress the immune system to help the body fight cancer, infection, and other diseases. The history of immunotherapy can be traced back to the late nineteenth century, but significant advances have been achieved in recent decades, stemming from a deeper understanding of immune regulation mechanisms. Several types of immunotherapies have been developed. Immunomodulatory molecular therapy, such as checkpoint inhibitors, cytokines, oncolytic viruses, or vaccines, aims to modulate endogenous natural immune systems. Cell-based immunotherapy, such as adoptive cell therapies using T cells or natural killer (NK) cells and stem cell therapies, infuses the recipient’s *ex vivo* expanded autologous cells to combat diseases. In certain cases, allogeneic cells may be used (i.e., hematopoietic stem cell transplants). Among these, immune checkpoint inhibitors [1] and cell-based therapies using tumor-antigen-specific T cell receptor (TCR)-transduced T cells [2, 3] and chimeric antigen receptor T (CAR-T) cells transduced with an antibody-derived single-chain variable region (scFv), which recognizes the cancer-associated antigen and activates an intracellular signaling domain [4, 5], have quickly become main therapeutic strategies, revolutionizing the field of oncology. Genetic engineering techniques have expanded to other cell types. These therapies have achieved durable clinical responses; however, their efficacies vary greatly, and sometimes life-threatening side effects have been observed [6, 7]. This variable response remains puzzling.

It is unsurprising that with the development of immunotherapies, interest in non-invasive imaging methods has increased. Immunomodulatory molecular therapies would benefit from visualization of the magnitude and extent of modulation induced in the immune microenvironment. In cell-based therapies, information on migration, activation, and expansion of the transferred cells could be important. Imaging such events would help address the failure in achieving the anticipated therapeutic effects and aid in developing new therapeutic strategies to obtain better outcomes. Various imaging methods and techniques have been investigated preclinically. Since each method has strengths and weaknesses, different imaging methods may be used depending on the immunotherapy strategy, cell type used, and treatment condition.

This review first provides a brief overview of various imaging methods for monitoring immunotherapies and then discusses more in detail clinically translatable imaging technologies with a focus on imaging cell-based immunotherapies. The review will conclude with an assessment of the current state of clinical translation.

## The Role of Imaging in Immunotherapy

Immunotherapies modulate the immune system to treat cancers or inflammatory diseases. It is critical that, in immunomodulatory molecular therapies, the aimed changes are induced in the target microenvironment, while in cell-based therapies, the transferred cells demonstrate migration to the target organ. For instance, CAR-T cells have enhanced cancer-antigen recognition capabilities that mediate cell killing, but to be effective, they must first infiltrate the tumor bed [4, 5]. Therapy failures can be ascribed to insufficient modification of the tumor microenvironment or under-delivery of therapeutic cells to the target. In the clinic, evaluation of immunotherapies relies on biopsies and blood sampling that are limited by their invasiveness and sampling errors. While in preclinical studies, harvesting tissue to analyze the induced changes is possible, longitudinal analysis is precluded.

Imaging can non-invasively visualize a magnitude of therapeutic effects, changes in cell distribution or metabolism, and migration, activation, expansion, and survival of therapeutic cells, all potential indicators of therapeutic outcome. For instance, the fraction of infused cells that trafficked to the target organ or tissues can help investigators estimate the number of cells needed to achieve the expected results. Pharmacokinetic information can be provided from dynamic or longitudinal imaging. Detection of off-target effects by imaging could predict potential side effects at early time points, increasing the opportunity for proper treatment. Overall, non-invasive imaging methods are powerful tools, allowing investigators to address reasons for treatment failure, optimize various parameters, and develop new immunotherapies.

## Overview of Imaging Methods for Immunotherapy

Various imaging technologies have been investigated for whole-body imaging of immunotherapies. Radionuclide imaging, such as single photon emission tomography (SPECT) and positron emission tomography (PET), and magnetic resonance imaging (MRI) have been used for both clinical and preclinical studies. Optical imaging such as bioluminescence imaging (BLI) and fluorescence imaging are widely used preclinically, but their application to humans is limited by the immunogenicity of non-human origin proteins that must be used and because of the very poor transmission of light through tissue, typically between a few millimeters to a few centimeters.

### Radionuclide Imaging

SPECT and PET can be used to image cells either by injecting a radiotracer specific for a biomarker expressed on the cell surface (*in vivo* labeling) or by labeling cells *ex vivo* and re-infusing into a recipient. Scintigraphy/SPECT agents indium-111 (^111^In)-oxine and technetium-99m (^99m^Tc)-hexamethylpropylene amine oxime (HMPAO) have been used nearly 50 years in imaging leukocytes and visualizing inflammation and infection or abscess in patients [8–10]. However, imaging tends to be slow, and relatively high radiolabeling doses are required for cell detection, which can cause radiotoxicity in the cells. Compared to SPECT, PET has higher sensitivity and provides better spatial and temporal resolution, and quantitation is more straightforward [11]. In immunoPET, positron emitters are conjugated to antibodies, antibody fragments (e.g., F(ab’)_2_, scFv), or engineered antibodies (e.g., minibodies, diabodies, bispecific antibodies) and infused for cell targeting. ImmunoPET can be designed to target various biomarkers, including cancer-related antigens, immune cell markers, and immune checkpoints [12, 13]. ImmunoPET imaging before and after an immunomodulatory molecular therapy can demonstrate the induced microenvironmental changes [13–15]. However, immunoPET cannot distinguish transferred cells from endogenous ones that express the same target biomarker, and thus, generally, cannot track therapeutic cells in cell-based therapies. For immunoPET to detect infused cells, use of cells introduced with a specific marker and a tracer targeting the marker would be required. The long circulation time and tissue pooling of the tracer increase background signals [16]. Specific and non-specific distribution of tracers and their clearance (e.g., liver, spleen, kidneys) could result in high background signals, hindering detection of cells. Antibodies/antibody fragments with high specificity are required for accurate imaging.

Visualizing the migration of infused cells can be achieved by labeling the cells with a radiotracer *ex vivo* before re-infusion. *Ex vivo* cell labeling methods achieve high signal-to-background ratios even with low cell labeling doses because only the cells infused have a radiotracer. Background signals can increase over time for reasons such as release of the tracer from the labeled cells. When cells undergo division, the label is passed over to daughter cells, halving the amount of tracer per cell [17], which also results in decreased signal-to-background ratios. Using SPECT, ^111^In-oxine-labeled tumor-infiltrating T cells (TILs) have been tracked over a week [18, 19]. ^99m^Tc-HMPAO-labeled autologous hematopoietic stem and progenitor cells (HSPCs) have been tracked in idiopathic dilated cardiomyopathy patients [20], but the short half-life of ^99m^Tc (6 h) severely limits the scanning time frame. Both ^111^In-oxine and ^99m^Tc-HMPAO are hydrophobic and passively enter the cells [21, 22]. Retention of ^111^In is thought to be mediated by transchelation of ^111^In to intracellular proteins. Similar intracellular protein binding of ^99m^Tc-HMPAO may occur. Conversion of HMPAO to a hydrophilic complex by reducing agents, such as glutathione, is thought to prevent release of the label. However, as this conversion is reversible, some ^99m^Tc-HMPAO, as well as free ^99m^Tc, will be released from the cells, causing background signal accumulation in the gastrointestinal and urinary tracts [10].

**Fig. 1.**
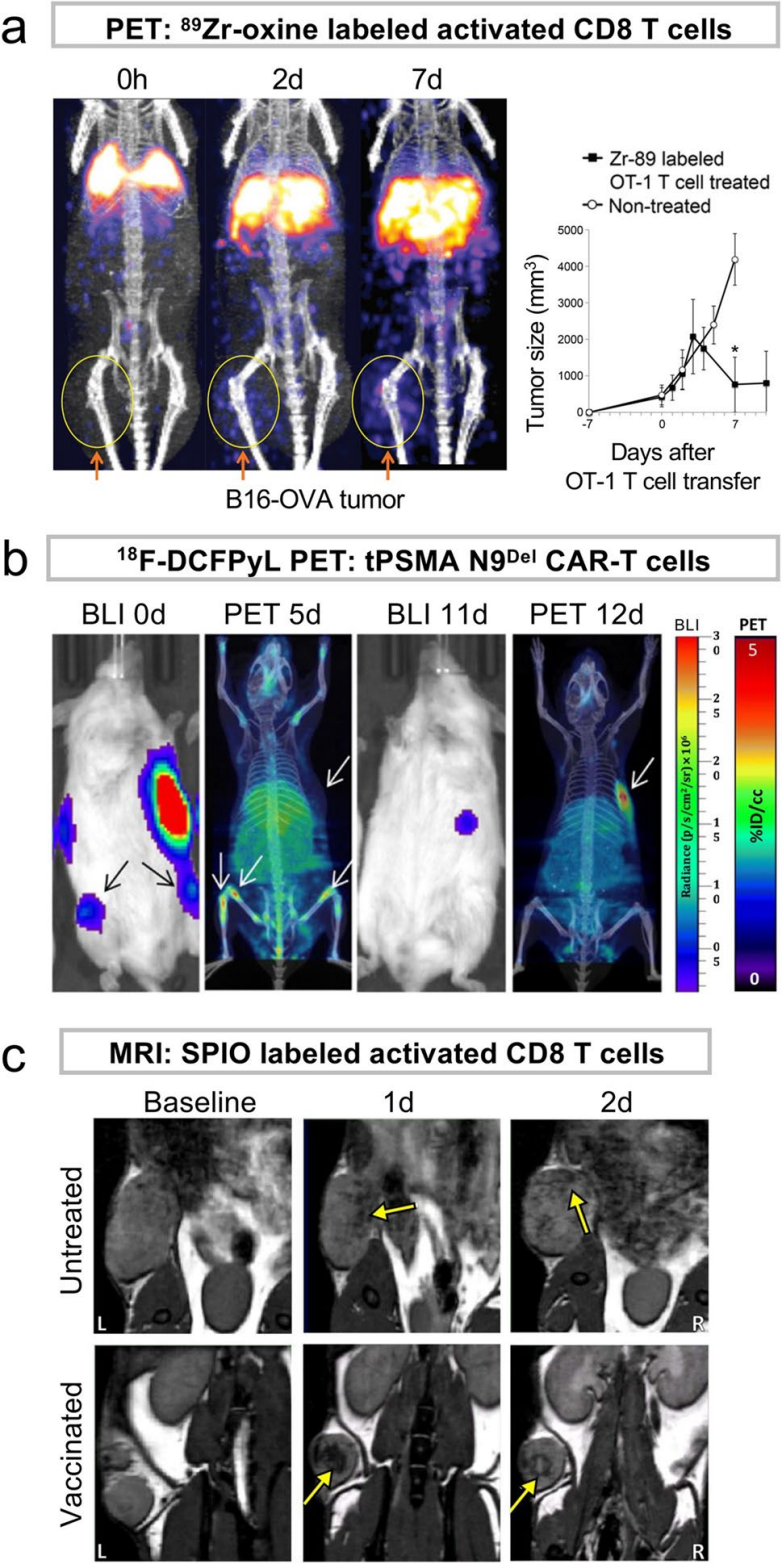
PET and MRI detection of ex vivo labeled and indirectly labeled adoptively transferred T cells targeting cancers. **a** PET/CT images of ^89^Zr-oxine-labeled OT-1 CD8 T cells accumulating in the B16-OVA melanoma tumor, which induced tumor regression. Adapted from [17]. **b** ^18^F-DCFPyL PET of CD19-tPSMA CAR-T cells infiltrating into local and metastatic Nalm6-eGFP-fLuc tumors. Tumor regression was shown by alternated BLIs, and changes in CAR-T cell accumulation were observed. Adapted from [51] with permissions from publisher. **c** MRI of SPIO-labeled CD8 T cells recruited to the C3 cervical cancer shown as hypo-intensity areas. The cells infiltrated deeper into the tumor in mice pre-vaccinated with cancer-specific peptide compared to untreated mice. Adapted from [42] with permissions from publisher.

To track cells by PET, 2-deoxy-2-[^18^F]fluoro-D-glucose (^18^F-FDG) has been used to label cells [23], but the half-life of ^18^F (110 min) limits the imaging window to a few hours. Because ^18^F-FDG incorporation depends on glucose uptake mechanism, ^18^F-FDG does not label metabolically inactive cells and is subject to efflux [23–25]. Copper-64 (^64^Cu, 12.7-h half-life)-pyruvaldehyde-bis(N4-methylthiosemicarbazone) (PTSM) has been used to track glioma cells and lymphocytes but rapidly releases ^64^Cu (22% remaining in 1 day), which leads to accumulation of free ^64^Cu in the liver [26], causing problematic high background in the abdominal area. Zircconium-89 (^89^Zr, 3.3-day half-life) has relatively low positron energy required for high-resolution imaging and lacks Auger electron emission [27]. ^89^Zr-oxine has been recently developed as an agent to label cells *ex vivo* [17, 28] and has successfully tracked various immune cell types for 1–2 weeks [17, 28–32] (Fig. 1a). Direct conjugation of ^89^Zr-deferoxamine-NCS to cell membrane has been applied to human mesenchymal stem cells (MSCs) [33].

Transfection/transduction of cells with a reporter gene that enables incorporation of a tracer allows for visualization of the cells after transfer [34, 35]. Because the reporter protein needs to be expressed in the cells via transcription/translation, only live cells incorporate the tracer. The reporter gene permanently integrated into the genome will be inherited by all subsequent daughter cells, allowing for long-term and repetitive imaging, without cell division-induced signal dilution [36]. In preclinical studies, reporter genes are often tailored to depict specific events. For example, a reporter gene might be placed under the control of a specific promoter so that expression of the reporter indicates activation of the promotor. Various reporter gene systems have been developed for SPECT/PET [36–38], MRI [39], and optical imaging (e.g., bioluminescent imaging, BLI) [34, 35]. Use of foreign reporter systems (e.g., herpes simplex virus type 1-thymidine kinase, HSV1-tk, and its variants) provides high signal-to-background ratios; however, clearance of tracers can still result in background signals [35]. SPECT/PET reporter imaging systems based on human endogenous molecules include sodium-iodide symporter (NIS, solute carrier family 5 member 5: SLC5A5), somatostatin receptor 2 (SSTR2), and prostate-specific membrane antigen (PSMA, Fig. 1b) [36–38, 40]. Most of them are compatible with multiple SPECT and PET radiotracers, including many clinically used tracers, adding flexibility to the choice of tracer depending on availability.

### MRI and MPI

MRI provides excellent anatomical resolution without ionizing radiation. MRI-based cell tracking most commonly uses superparamagnetic iron oxide nanoparticles (SPIOs), which are phagocytosed by the cells, and have been performed in several preclinical models, including CD8 T cell targeting cancer [41, 42] (Fig. 1c). However, SPIOs cause a distortion in the magnetic field, which leads to signal loss often greater in size than the actual accumulation, making cell distribution unquantifiable [43]. Detection of SPIO-labeled cells of unknown distribution can be difficult due to background signals, and it is challenging to acquire whole-body imaging in a reasonable time period. MRI reporter gene strategies have also been explored, such as the transferrin receptor and ferritin system, but the sensitivity is too low for clinical applications [39].

A method of using ^19^F-perfluorocarbon (PFC)-based cell labeling agents to track cells with MRI has been developed recently. ^19^F-PFCs can be used for both *ex vivo* and *in vivo* cell labeling. The MRI unit is tuned to the resonance frequency of ^19^F, but additional ^1^H-MRI is acquired for anatomical localization. ^19^F-MRI shows high specificity for labeled cells due to the lack of natural ^19^F in the body, which is quantifiable. Cells such as dendritic cells (DCs) [44], T cells [45], and NK cells [46] have been tracked preclinically and clinically. However, specialized ^19^F detection coils are required for imaging, and the sensitivity is low. ^19^F-PFCs also rely on phagocytosis for labeling, and thus non-phagocytic small cells (e.g., lymphocytes) are difficult to label.

Magnetic particle imaging (MPI) is a new imaging technology that also utilizes SPIOs. Unlike regular MRI, signals from SPIOs have fewer artifacts with MPI and can be quantitated. Combination with CT or MRI is performed for anatomical imaging. Tracking SPIO-labeled human MSCs [47] and T cells [48] administered to mice has been performed. The process, however, is slow and cumbersome and requires raster-like acquisitions through the body.

### Optical Imaging

BLI and fluorescence imaging are widely used in preclinical studies due to their relative simplicity and convenience. In BLI, luciferin is injected and is converted into light by the enzyme luciferase in cells that have been transfected/transduced with a luciferase gene. BLI allows for monitoring proliferation, migration, and death of the cells for relatively long term in preclinical models [49]. Luciferase-expressing tumors are often used to monitor therapeutic effects (Fig. 1b), and NK-T and T cells have been tracked in adoptive immunotherapy models [49–51]. A membrane-anchored form of the Gaussia luciferase shows higher signal compared to D-luciferin-based firefly luciferase and coelenterazine-based Renilla and the original Gaussia luciferases in detecting T cells and visualized CAR-T cells targeting tumor [52]. While signals from coelenterazine-based luciferases, including membrane-anchored Gaussia luciferase, start to “fade” within a few minutes, firefly luciferase and a recently developed vargulin-based membrane-anchored Cypridina luciferase show stability over 15 min [53]. Multiplex BLI is possible by using different luciferase/substrate combinations. Detection of less than 10 cells has been reported [54, 55]. However, because luciferin and luciferase are foreign proteins to humans, and therefore immunogenic, BLI is not applicable for humans. Fluorescent imaging with near infra-red probes has been used for tracking *ex vivo* labeled cells such as T cells and neutrophils [56, 57]. Optical imaging is subject to tissue absorption of light, and whole-body quantification is not possible [58]. Confocal and intravital microscopies can be used to track cells on a small scale. These microscopic analyses, although limited to the small observation area, allow for direct visualization and characterization of *in vivo* cell behaviors and cellular processes with spatiotemporal dynamics at a single-cell level [59].

## Emerging Clinically Translatable Methods for Tracking Cell-Based Therapy

Cell-based immunotherapies are amenable to direct cell labeling procedures at the end of *ex vivo* cell expansion before infusion of the cells to patients. Reporter gene transduction can be performed during the cell expansion. In general, PET is becoming the most well-known method of imaging these therapeutic cells. In the following section, we discuss details of emerging clinically translatable imaging methods for cell-based therapies: direct *ex vivo* cell labeling and indirect cell labeling via reporter genes for PET and MRI.

### Ex Vivo Cell Labeling Methods for Tracking Cells with PET

Two groups have independently developed ^89^Zr-oxine for tracking cells using different methods [17, 28]. Slight modifications to the methods [30] and more detailed optimization of synthesis conditions for generating a good manufacturing practice (GMP)-compatible kit have been performed [60]. An on-cartridge synthesis of ^89^Zr-oxine, as well as ^64^Cu-oxine and ^64^Cu-tropolone, has been explored [61].

**Fig. 2.**
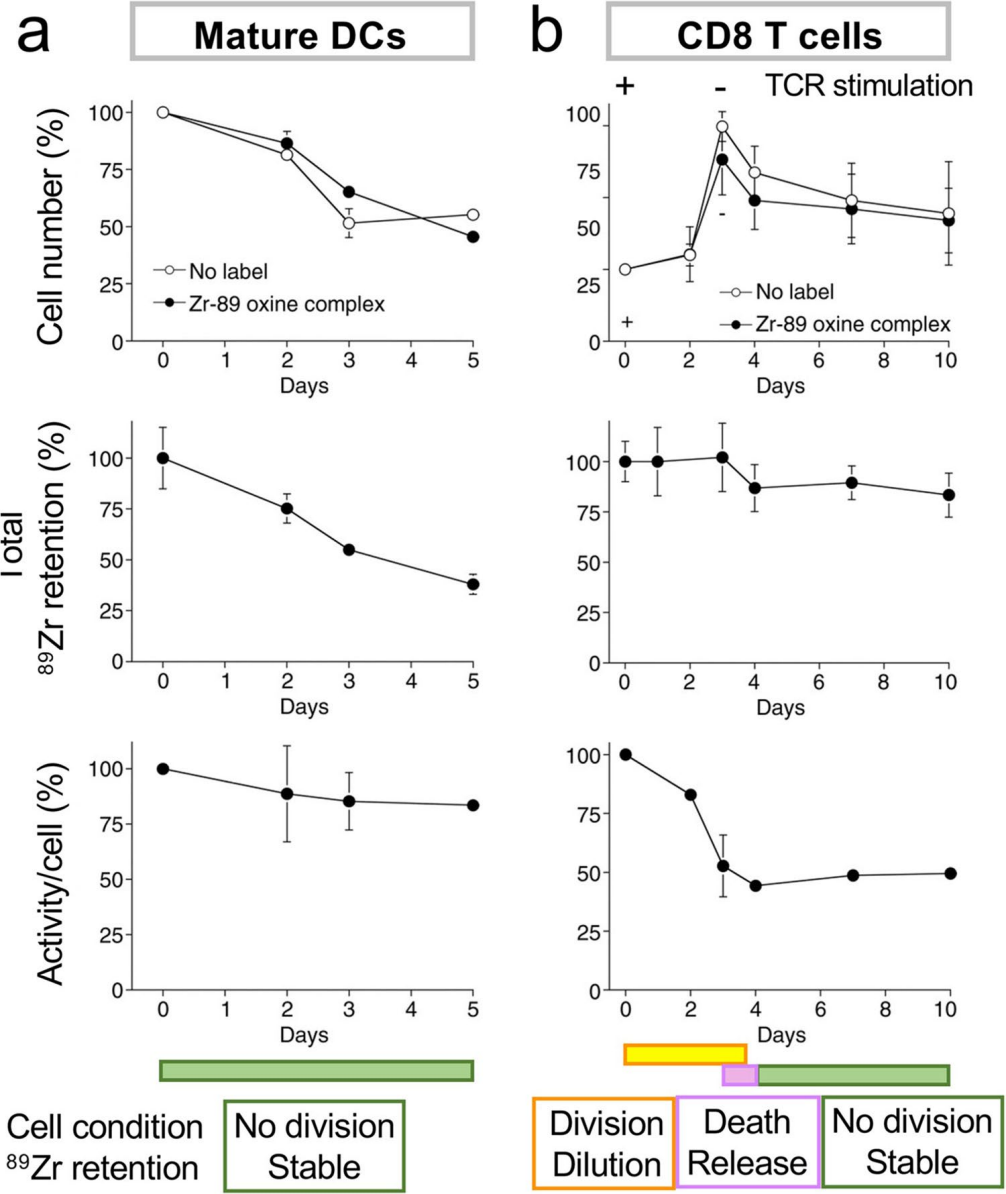
Retention of ^89^Zr affected by the condition of ^89^Zr-oxine-labeled cells: non-deviding, dividing, and death. **a** Mature DCs do not divide. ^89^Zr-oxine-labeled mature DCs survived similar to non-labeled DCs and maintained the specific activity. **b** ^89^Zr-oxine-labeled CTLs underwent TCR-induced proliferation followed by cell death during the contraction phase, similar to non-labeled CTLs. ^89^Zr activity per cell decreased during the cell division, and ^89^Zr was released by the cell death. All figures were adopted from [17].

**Fig. 3.**
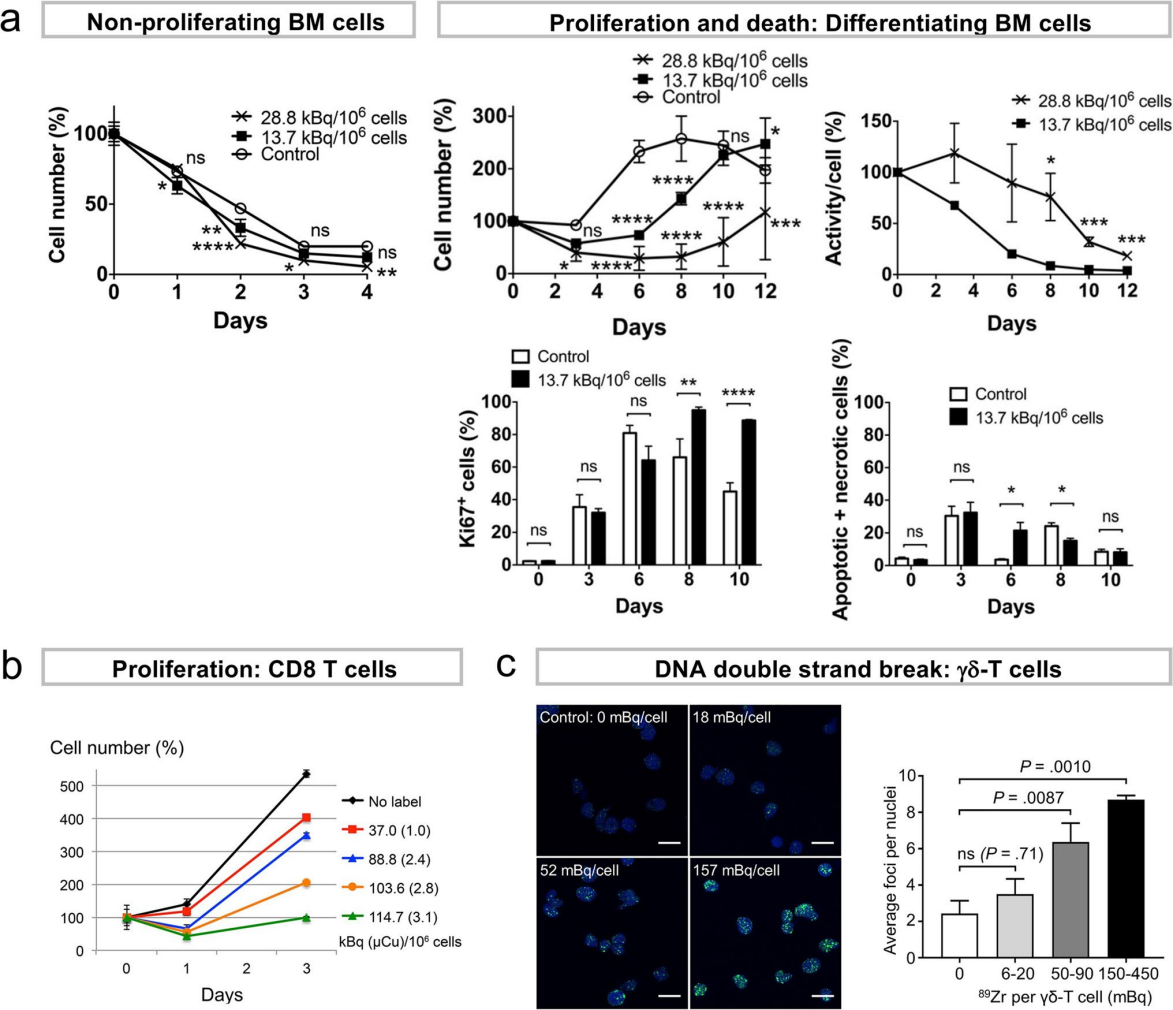
Optimization of ^89^Zr-oxine labeling dose to minimize radiotoxicity. **a** ^89^Zr-oxine-labeled bone marrow (BM) cells followed comparable decline of viability to non-labeled control in culture without cytokines. In culture with GM-CSF to induce differentiation, BM cells showed delayed proliferation and were mixture of proliferating (Ki67^+^) and apoptotic/necrotic cells. As a result, calculated specific activity declined in cells labeled at a lower labeling dose and was maintained at earlier time points at a higher labeling dose. Adapted from [64]. **b** ^89^Zr-oxine-labeled activated CTLs cultured in IL-2 showed dose-dependent suppression of proliferation. Adapted from [17]. **c** $\gamma \delta$-T cells labeled with ^89^Zr-oxine showed dose-dependent increase of DNA double-strand breaks indicated by $\gamma$-H2AX foci (green) in the nuclei (blue). Adapted from [31] with permission from the publisher.

^89^Zr-oxine is lipophilic and permeates the cell membrane, enabling cell labeling even at 4 °C when active cellular uptake is absent [17]. Because of this labeling mechanism, any cell types can be labeled independent of its surface biomarkers or cellular condition. It is likely that ^89^Zr-oxine labeling follows a similar mechanism as that of ^111^In-oxine. After entering a cell, exchange of ^89^Zr from oxine to intracellular proteins likely takes place, oxine being exported from the cell while ^89^Zr is retained [62]. The differences in the ^89^Zr-activity incorporation rate by cell type reported [17] may result from differences in the amounts of intracellular proteins available for ^89^Zr binding. To label cells at optimal doses, pre-evaluation of incorporation rate is required. ^89^Zr-oxine-labeled cells, especially non-dividing cells (e.g., matured DCs), retain ^89^Zr well over multiple days (Fig. 2a). As with other *ex vivo* cell labeling agents, intracellular ^89^Zr dilutes as the cells divide [17] (Fig. 2b). After cell death, ^89^Zr is released from the cells (Fig. 2b) [17], presumably due to the release of ^89^Zr-bound intracellular proteins through the compromised membrane. Evaluation of ^89^Zr retention in proliferating cells is complicated by the combination of cell division and death (Fig. 3a, b). For instance, if a portion of the labeled cells is divided (dilution of label) but a portion of them died (loss of label), a condition could occur in which calculated activity per cell would decrease, despite the retention of ^89^Zr in viable non-divided and divided cells. Difference between release of label from live cells (label instability) and that from dead cells (compromised cell integrity) should be noted, especially when discussing the efflux. The direct conjugation of ^89^Zr-deferoxamine-NCS to cellular membrane eliminates release of ^89^Zr [33]. However, this could be a double-edged sword, causing transfer of all ^89^Zr to phagocytes after cell death and persistent ^89^Zr signal in the absence of transferred cells. The conjugation process could affect viability in sensitive cells, and alteration of cellular function by the membrane protein modification and possible induction of downstream signaling events remain as concerns.

### Radiotoxicity in Ex Vivo Cell Labeling

When tracking cells by imaging, it is critical that the labeled cells behave in the same manner as non-labeled cells. One of the easiest but sensitive tests of evaluating cellular toxicity is documenting live cell number changes under conditions of heightened cell proliferation. Since cytotoxicity may not be immediate, sufficient time must be allowed to elapse for detection. Documenting live cell percentage potentially underestimates the cell death occurring over multiple days, as fragmented dead cells are not counted. Dividing cells are more sensitive to radiation than non-dividing cells and thus could exhibit cytotoxicity at the low labeling doses that are non-toxic in non-dividing cells (Fig. 3a). A ^89^Zr-oxine dose escalation study using activated CD8 T cells suggested requirement of a specific activity of less than 37 kBq/10^6^ cells for minimizing the toxicity [17] (Fig. 3b). Another study using $\gamma \delta$-T cells indicated that 6–20 kBq/10^6^ cells was not toxic, whereas 50–90 kBq/10^6^ cells abrogated proliferation and showed DNA double-strand breaks [31] (Fig. 3c). Comparison of DNA damage occurrence between ^111^In-oxine and ^89^Zr-oxine, performed on accumulated data using different activity doses and numbers of white blood cells for each agent and sample, demonstrated that ^89^Zr-oxine caused slightly more DNA damage than ^111^In-oxine when compared to non-labeled control [60]. Previous reports have shown that ^111^In-oxine labeled HSPCs lose proliferative function [63], while ^89^Zr-oxine-labeled bone marrow cells show delayed proliferation but maintain differentiation capability [64] (Fig. 3a). As of yet, no side-by-side comparison of radiotoxicity between ^111^In-oxine and ^89^Zr-oxine at comparable doses exists. However, considering extremely low doses of ^89^Zr-oxine required for imaging, labeling immune cells with ^89^Zr-oxine can be performed without significantly affecting cellular function. For instance, ^89^Zr-oxine labeling of CD8 T, CAR-T, and NK cells does not affect cellular viability, proliferation, cytokine production, or cytotoxicity [17, 29, 30]. Accumulation of labeled CD8 T cells in the tumor and resulting tumor shrinkage have been observed [17] (Fig. 1a). Labeled DCs can be activated and present antigen to T cells [17]. Chemotaxis is maintained in labeled bone marrow cells [64, 65] and in eosinophils [32]. These studies suggest that the optimal labeling doses that provide sufficient PET detection while minimizing radiotoxicity are approximately 11–44 kBq/10^6^ cells, depending on type and condition of the cells.

### Limits of Radiolabeled Cell Detection by PET

**Fig. 4.**
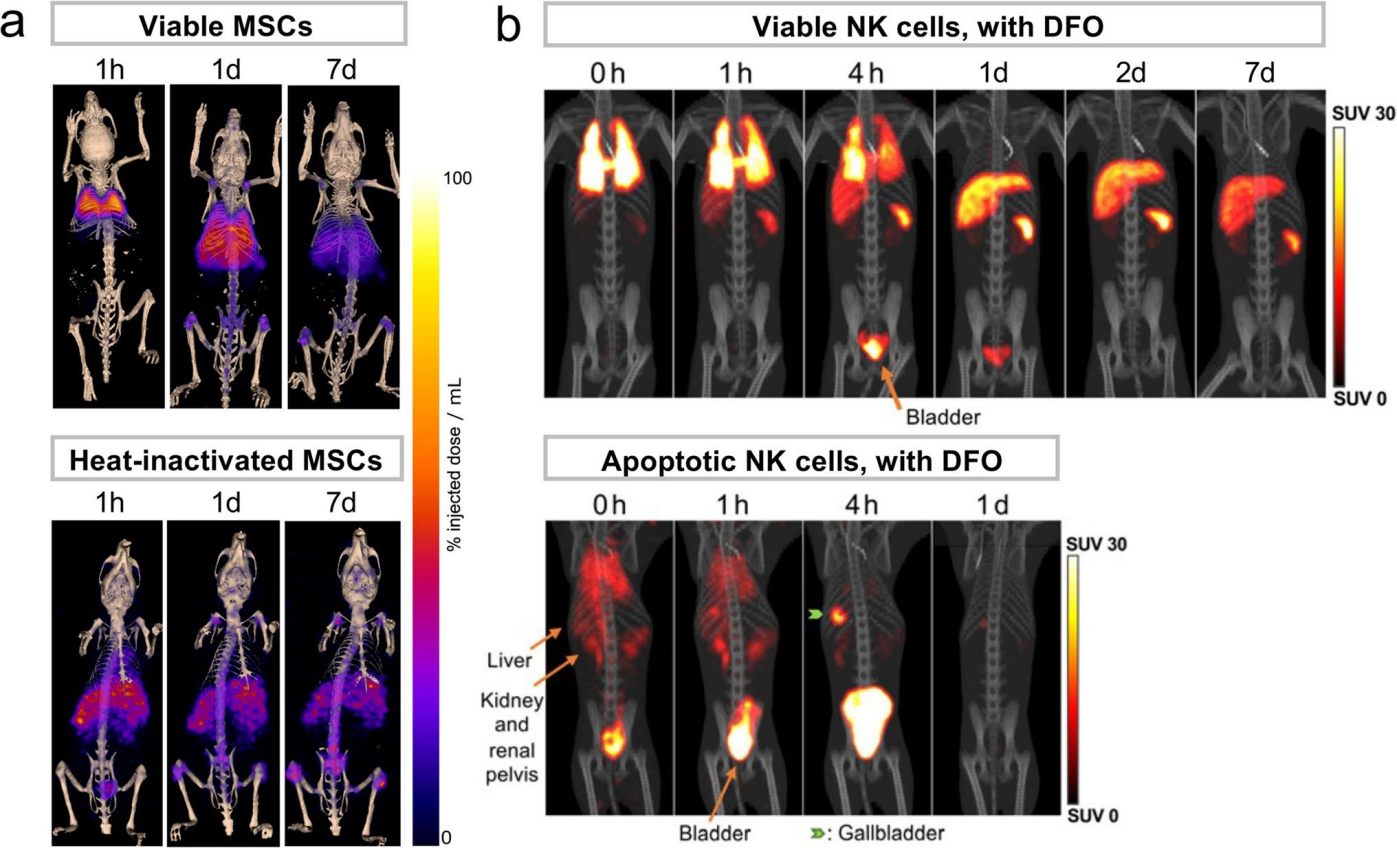
In ^89^Zr-oxine-labeled cell tracking by PET, deferoxamine (DFO) infusion prevents bone uptake of free ^89^Zr released from dead cells. **a **^89^Zr-oxine-labeled allogeneic MSCs expressing TRAIL (MSCTRAIL) were intravenously administered. Cells distributed in the lungs. ^89^Zr-signals distributed in the liver, spleen, and bones after 1 day, but no indication of live cells was found in these organs. Injection of heat-inactivated dead MSCTRAIL showed ^89^Zr activity in the liver and spleen, where dead cell can be taken up, and bones, where free ^89^Zr is known to bind. Adapted from [67] with permission from the publisher. **b** ^89^Zr-oxine-labeled autologous NK cells distributed in the lungs initially and then in the liver and spleen. DFO infusion enhanced renal clearance of free ^89^Zr released from dead cells. DFO effectively prevented bone uptake of ^89^Zr after apoptotic NK cell administration. Adapted from [29].

*Ex vivo* labeling methods provide excellent sensitivity and signal-to-background ratios, requiring only extremely low labeling doses, which contribute to minimize radiotoxicity. It allows detection of relatively small changes of cell distribution induced after cell infusion [32, 65], but the signals dilute by cell divisions. In ^89^Zr-oxine PET, although ^89^Zr is stably retained in viable cells, free ^89^Zr released from the dead cells may be taken up in bone matrix hydroxyapatite [17, 64, 66, 67] (Fig. 4a). Infusion of deferoxamine to chelate and excrete the free ^89^Zr from the kidneys has been proven effective in preventing bone uptake of ^89^Zr [29, 64] (Fig. 4b). Deferoxamine could also minimize transfer of ^89^Zr to phagocytes by quickly chelating the ^89^Zr before phagocytosis takes place. Based on NK cells and HSPCs tracking studies performed in rhesus macaque using a clinical PET/CT scanner, as low as approximately 55 kBq/kg dose of ^89^Zr-oxine-labeled cells (approximately 3 × 10^6^ cells/kg dose with cells labeled at 18.5 kBq/10^6^ cells) could be imaged with high quality and have their migration quantitated [29]. Also using clinical PET/CT and PET/MRI scanners, 3.3 × 10^4^/cm^3^ Jurkat cells labeled with ^89^Zr-oxine at 15.4 kBq/10^6^ cells were detected [68].

### Reporter Gene Imaging for Cell Tracking with PET

An alternative to *ex vivo* cell labeling is reporter gene imaging. Reporter gene technique can selectively visualize live cells at a time remote from the cell transfer. Because images are acquired after sufficient clearance of unbound tracers and the imaging interval is determined by decay of the radiotracer, radiotracers with rapid clearance and short radioactive half-lives are favored. Still, detection of rapid cell distribution changes (e.g., within hours) induced by exogenous stimuli could be challenging. Unlike *ex vivo* cell labeling, it is difficult to evaluate radioactivity doses incorporated into the target cells *in vivo*, and thus controlling and assessing the radiotoxicity are very difficult. *In vitro* radiotoxicity assays do not mimic the *in vivo* condition where the radiotracer extravasate to reach the cells in tissues while constantly being cleared from the body. The need for reporter gene insertion in the cell genome has been a stumbling block for its clinical application. However, increased clinical use of genetically engineered cells (e.g., CAR-T cells) has lowered the barrier for introducing a reporter gene. For the cells that do not require genetic engineering (e.g., TILs, stem cells), *ex vivo* cell labeling methods would still be the first choice.

**Fig. 5.**
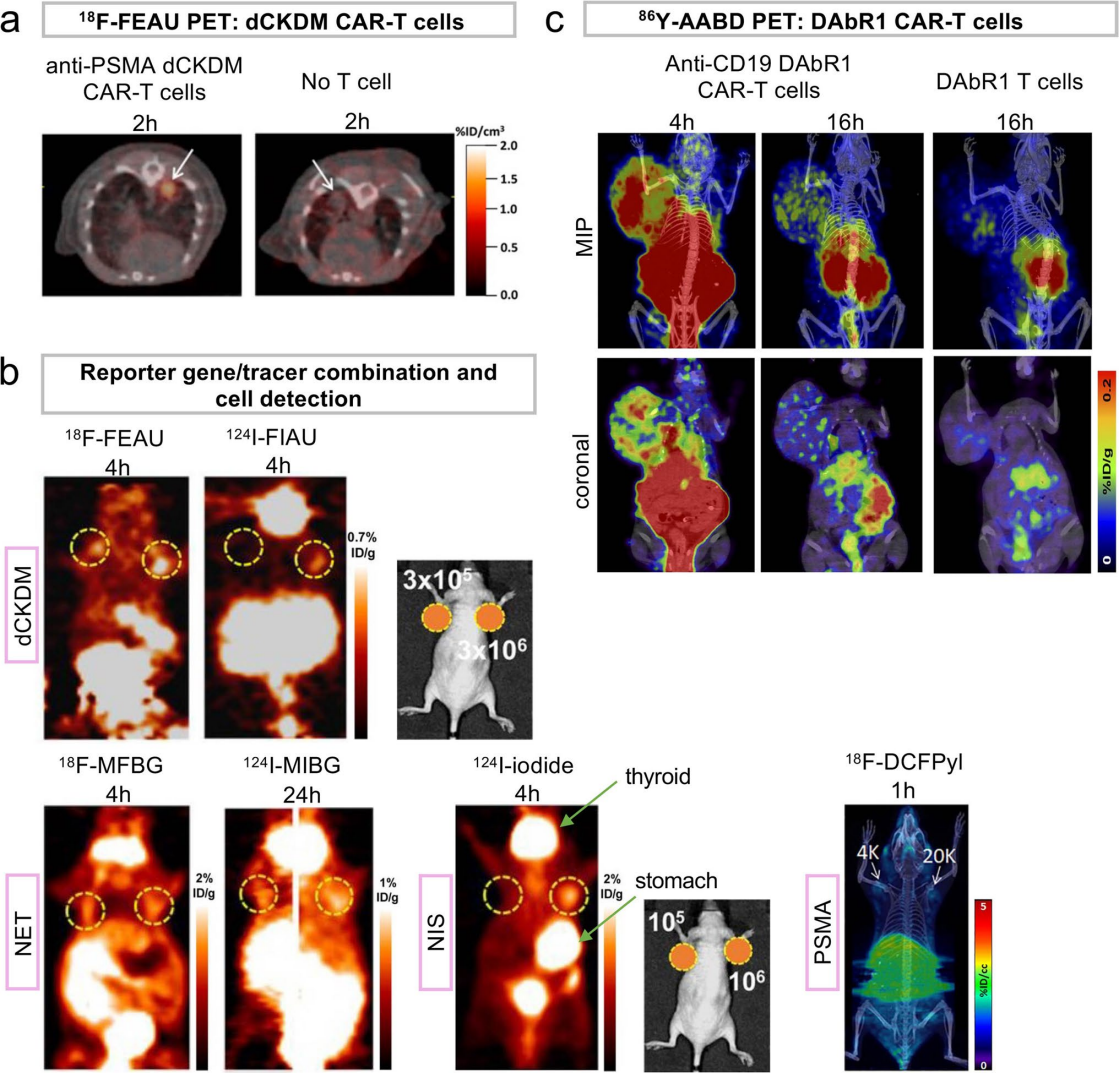
Examples of reporter gene PET imaging of adoptively transferred CAR-T cells targeting cancers and cell detection sensitivity. **a** ^19^F-FEAU PET/CT images at 6 h after anti-PSMA dCKDM CAR-T cell transfer and 2 h after tracer injection show high tracer accumulation in pulmonary PC3/hPSMA tumors. Without T cell transfer, ^19^F-FEAU did not accumulate in tumors. Adapted from [71]. **b** The combination of reporter gene and tracer affects target cell detection sensitivity. Gastrointestinal clearance of the tracer and physiological uptake to normal organs could make detection of the cells in the proximity (e.g., abdominal area) difficult. Adapted from [51, 72]. **c** ^86^Y-AABD PET/CT images at 4 and 16 h after injection depicting accumulation of anti-CD19 CAR-T cells co-transduced with DAbR1 at Nalm-6 tumor. No uptake above background at tumor site is noted after DAbR1 T cell administration. Adapted from [80].

HSV1-tk has been used in CAR-T cells targeting IL-13 zetakine (receptor $\alpha$2), whose expression within the central nervous system is restricted to glioma cells, infused to post-operative cavity in recurrent glioma patients. 9-(4-^18^F-Fluoro-3-[hydroxymethyl]butyl)guanine (^18^F-FHBG) PET showed some accumulation of the T cells to the tumors [69]. However, immunogenicity of the viral protein HSV1-tk prevents general use of this system in humans. Human mitochondrial thymidine kinase 2 (TK2), deoxycytidine kinase (dCK), and their double mutants (e.g., TK2DM, dCKDM) are detected by 2′-fluoro-2′ deoxy-1-β-D-arabinofuranosyl-5-[^124^I]iodouracil (^124^I-FIAU), 2′-[^18^F]fluoro-5-ethyl-1-b-D-arabinofuranosyluracil (^18^F-FEAU), or 1-(2′-[^18^F]fluoro-5-methyl-β-L arabinofuranosyl)uracil (^18^F-L-FMAU), which are also used by HSV1-tk. dCK has been used to track hematopoietic stem cell transplants and anti-PSMA-CAR-T cells targeting lung tumor in mouse models using ^18^F-L-FMAU and ^18^F-FEAU  (Fig. 5a), respectively [70, 71]. dCK detects around 3 × 10^5^ subcutaneously injected T cells using ^18^F-FEAU (Fig. 5b) [72].

Cell surface molecule-based human reporter gene systems include NIS that is compatible with multiple SPECT tracers (e.g., ^99m^Tc-pertechnetate, ^123^I) and PET tracers (e.g., ^124^I, ^18^F-tetrafluoroborate), SSTR2 with ^68^Ga-DOTA-D-Phe^1^-Tyr^3^-octreotate (^68^Ga-DOTATATE, DOTA: 1,4,7,10-tetraazacyclododecane-N,N′,N″,N′-tetra-acetic acid) and ^68^Ga-DOTA-D-Phe^1^-Tyr^3^-octreotide (^68^Ga-DOTATOC) for PET, norepinephrine transporter (NET, SLC6A2) with ^124^I-metaiodobenzylguanidine (^124^I-MIBG) for SPECT and ^18^F-meta-fluorobenzylguanidine (^18^F-MFBG) for PET, and PSMA with 2-(3-{1-carboxy-5-[(6-[^18^F]fluoro-pyridine-3-carbonyl)-amino]-pentyl}-ureido)-pentanedioic acid (^18^F-DCFpyI, Fig. 1b). These tracers have been used in the clinic, some for a long time, for detecting endogenous lesions (e.g., ^68^Ga-DOTATATE and ^68^Ga-DOTATOC to detect neuroendocrine tumors [73]). ^68^Ga-DOTATOC has been approved by the US Food and Drug Administration in 2019 and ^18^F-DCFpyl followed in 2021. ^18^F-tetrafluoroborate for NIS has been evaluated in healthy human volunteers [74] and thyroid cancer patients [75]. NIS does not internalize upon ligand binding but is physiologically expressed in organs such as thyroid, salivary/lacrimal glands, and stomach. SSTR2 is expressed in the kidneys, gastrointestinal tract, and hematopoietic cells. Internalization of SSTR2 and reported negative impact on immune cell function by an agonist are concerns [76].

NIS [77], SSTR2 [78], NET [79], and a PSMA variant tPSMA^N9Del^ engineered to prevent internalization and intracellular signaling [51] have been used to track T cells, including CAR-T cells, in mouse models. NIS has been reported to detect 3 × 10^3^ CAR-T cells *in vitro* with ^18^F-tetrafluoroborate PET [77] and 1.5 × 10^4^ subcutaneously injected cells *in vivo* with ^99m^Tc-pertechnetate SPECT [40]. Sensitivity of SSTR2 has been estimated to be 4 × 10^6^ Jurkat cells/cm^3^ tumor with ^68^Ga-DOTATOC [78]. NET has been reported to detect < 10^5^ subcutaneously injected T cells with ^18^F-MFBG [72] and PSMA to detect 2-3 × 10^3^ CAR-T cells incubated with ^18^F-DCFpyI *in vitro* (Fig. 5b) [51, 77]. Again, *in vitro* tracer incorporation assays and incorporation to subcutaneously injected cells do not accurately represent the *in vivo* tracer delivery and subsequent cellular uptake in physiological conditions. Possibly, real detection limits are higher in cell numbers than *in vitro* assay results and lower than results obtained from subcutaneously injected cells.

DOTA-antibody reporter gene 1 (DAbR1) is a new reporter gene system that introduces murine anti-DOTA scFv fused to human IgG4 CH2-CH3 and CD4 transmembrane domain [80]. This scFv forms a covalent bond with the acrylamide group of (*S*)-2-(4-acrylamidobenzyl)-DOTA (AABD) that can be conjugated with yttrium-86 (^86^Y, half-life 14.7 h) for imaging. CD19-CAR-T cell targeting to subcutaneous tumor has been demonstrated in mice (Fig. 5c). Consideration of scFv humanization is required for clinical application of DAbR1. The gastrointestinal tract clearance of ^86^Y-AABD, in addition to kidneys, causes high non-specific signals, making detection of the cells in the abdominal lesion difficult.

Various reporter gene tracers show gastrointestinal tract clearance (e.g., ^18^F-MFBG, ^18^F-FIAU, ^18^F-FEAU), which could be problematic in assessing whole-body cell distribution, such as analyzing memory T cell distribution or addressing unforeseen side effects from the therapeutic cells. Because of the differences in the normal organ distribution, clearance, and radioactive decay among the tracers, selection of the reporter gene/tracer combination affects image quality and cell detection sensitivity [72]. Of note, some of the reporter genes can become suicide systems by selecting a therapeutic counterpart of the tracers, such as lutetium-177 (^177^Lu)-DOTATATE for SSTR2, that kills the cells expressing the genes *in vivo* when transferred cells are no longer needed or cause problematic side effects. For a full review of reporter gene PET/SPECT imaging systems, including non-human systems, see references [36–38].

### *Ex Vivo* Cell Labeling Methods for Tracking Cells with MRI

SPIO-based cell tracking has been performed in DCs implanted intranodally [81, 82] and neural stem cells implanted in regions of brain trauma [83, 84]. Because of difficulty in detecting the labeled cells throughout the body, the clinical application seems to be more focused on image-guided local cell injections and tracking.

**Fig. 6.**
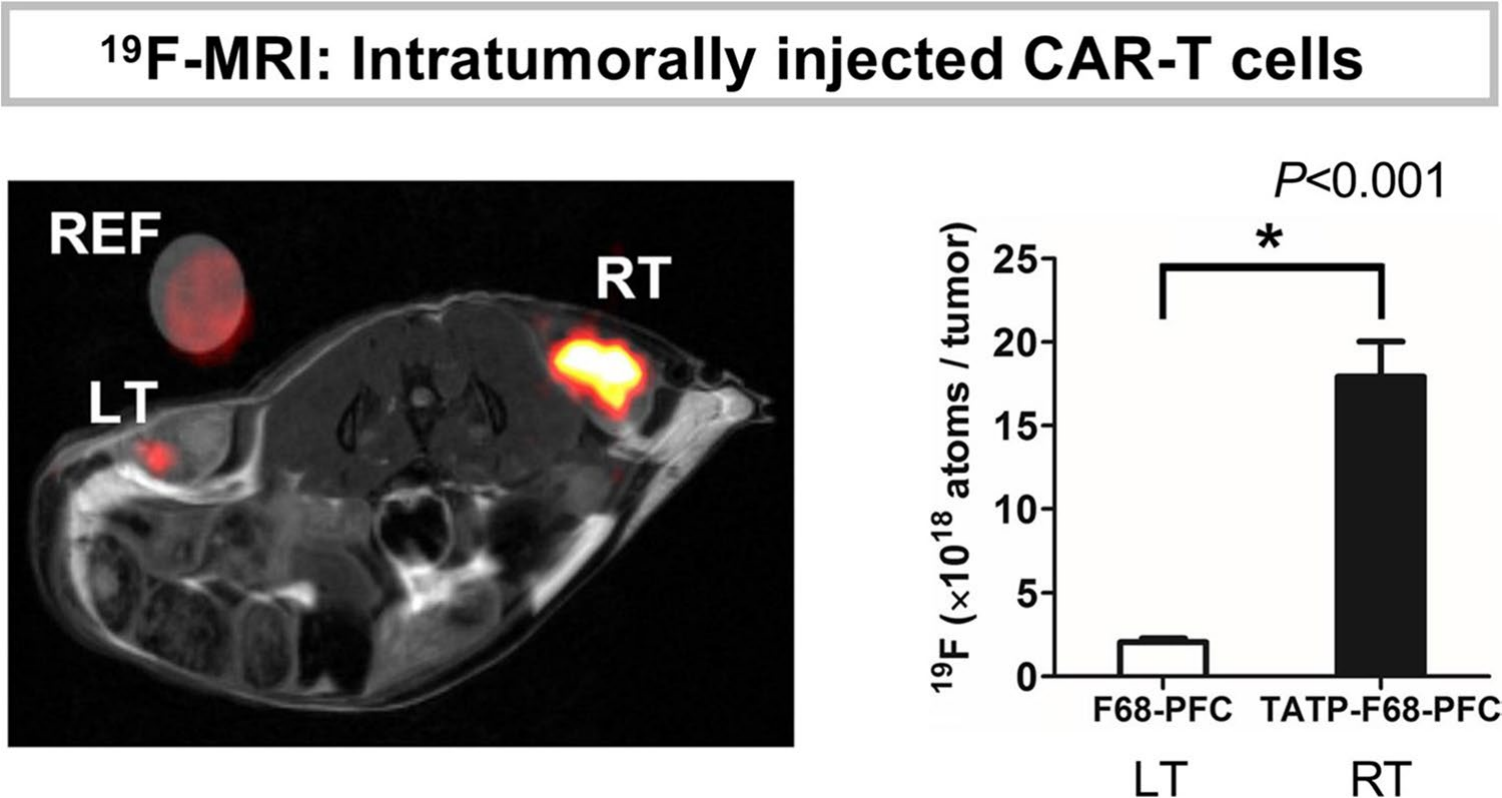
^19^F-MRI signal enhancement in cell-penetrating peptide TAT-PFC-labeled CAR-T cells. A mouse with bilateral EGFP-expressing gliomas received intratumoral injection of anti-EGFR CAR-T cells labeled with either F68-PFC (control, left: LT) or TATP-F68-PFC nanoemulsions (right: RT). An external capillary reference (REF) consists of 1:20 dilution of F68-PFC in agarose. Combined ^19^F (hot-iron) and ^1^H (grayscale) MRI images. TAT-F68-PFCs showed ~ eightfold in vivo apparent ^19^F atoms increase compared to control. Figures adapted from [87] with permission from the publisher.

^19^F-PFC-based cell labeling agents for ^19^F-MRI include perfluorooctyl bromide (PFOB), perfluoro-15-crown-5-ether (PFCE), and perfluoropolyether (PFPE), which are biologically inert, highly stable, and non-toxic [85]. A clinical trial has been conducted to evaluate intradermally delivered autologous DC vaccines. ^19^F-MRI detected 1 × 10^7^ cells but not 1 × 10^6^ cells, indicating an estimated DC detection sensitivity of approximately 10^5^ cell/voxel [44]. In general, sensitivity is affected by multiple factors such as the PFCs used, the cell type, image acquisition methods, and MRI configuration, ranging 10^3^–10^5^ cells per voxel [86]. To augment ^19^F incorporation within cells, cell-permeating transactivating transcription sequence (TAT) peptide-conjugated PFCs have been developed. Using TAT achieved > eightfold increase in ^19^F incorporation in CAR-T cells, resulting in significantly higher ^19^F-MRI signals [87] (Fig. 6). Generally, using transfection agents or larger size PFCs increases cellular uptake of ^19^F, but these agents (e.g., > 500 nm PFCs) can induce undesired activation of the cells [88].

### Multimodal Cell Tracking Methods

Multimodal cell tracking methods take advantage of different imaging modalities compensating for weaknesses. ^64^Cu-SPION is a PET-MRI multi-modal imaging nanoparticle that benefits from the high sensitivity of PET and the detailed anatomical information of MRI [89]. ^64^Cu-SPION-labeled CD19-specific CAR-T cells showed cytotoxic action against target lymphoma cells *in vitro*, although at a lower level than the unlabeled CAR-T cells [89]. A first-in-human clinical trial has been performed in CAR-T cells [90]. ^19^F-PFC containing radiometal chelate fluorous hydroxamic acid that captures ^89^Zr has visualized inflammatory lesions in mice, including an experimental inflammatory bowel disease and a periphery of tumors, presumably through phagocytosis by macrophages, both in PET and ^19^F-MRI [91]. Although the reported work is to detect endogenous macrophages, this agent holds promise for an application to *ex vivo* labeling of phagocytes in the context of cell-based immunotherapy.

## Considerations for Cell Tracking by Cell Type

### T Cells and NK Cells

T cells rapidly proliferate upon recognition of the nominal antigen, but proliferating T cells are especially sensitive to irradiation. Toxicity of ^111^In-oxine to labeled T cells has been reported [21]. Variance between the radioactive dose requirement for detection and radiotoxicity may limit the application of ^111^In-oxine for imaging T cells. Labeling expanded NK cells presents similar difficulties. In contrast, ^89^Zr-oxine has shown promise for tracking these cells [17, 29–31]. *Ex vivo* labeling with ^19^F-PFC for MRI has also been performed in T cells [87]. In either *ex vivo* cell labeling method, the signal strength in the target (e.g., cancer) reflects the labeled cells homed to the target, but not the cell expansion at the target. These methods will suffice to evaluate the homing property of the cells, such as cells engineered to enhance homing. However, if the goal is to evaluate the therapeutic efficacy of the cells, reporter gene imaging will more accurately reflect the cell number. Reporter gene imaging could track long-lived memory T cells differentiated from the transferred T cells and their responses to the recurrent tumors. It has been considered that NK cells do not differentiate into memory cells. However, accumulating evidence suggests the presence of a subset of NK cells that possess antigen-specificity and also a memory or memory-like NK cell subset [92].

### DCs

DCs, as they differentiate and mature, upregulate the major histocompatibility complex or human leukocyte antigen molecules and co-stimulatory molecules such as CD80, CD86, and CD40. The ability of DCs to present antigens to T cells differs by their maturation status. When they mature, they stop proliferation. In preclinical studies, careful selection of immature or mature DCs is needed. As DCs are relatively resistant to irradiation, PET or SPECT is a good modality for whole-body tracking of DCs. The phagocytic nature of DCs also enables *ex vivo* labeling with SPIOs and ^19^F-PFCs for MRI.

### Monocytes and Macrophages

Monocytes and macrophages are increasingly popular clinical candidates for cell-based immunotherapy due to their plasticity and functionality spectrum. Monocytes exhibit anti-cancer cytotoxicity in the presence of interferons and have been used in the treatment of patients with peritoneal metastatic ovarian cancer [93]. Macrophages can polarize into two opposite phenotypes, M1-type (pro-inflammatory, anti-cancer) vs M2-type (anti-inflammatory, pro-cancer). Reprogramming polarized macrophages into the desired type has been investigated for cell-based immunotherapy [94]. Monocytes/macrophages are highly phagocytic and incorporate SPIOs and ^19^F-PFCs for MRI [95]. As monocytes are prone to stimulations and their activation induces quick differentiation to macrophages, care should be taken during the labeling process.

### HSPCs

Labeling HSPCs with ^89^Zr-oxine and ^19^F-PFCs has shown to delay proliferation after labeling and slightly reduce viability, respectively, but retain multipotency [64, 96]. Minimum labeling doses must be selected when monitoring HSPCs. Trafficking of HSPCs in various recipient conditions has been successfully studied by ^89^Zr-oxine PET [64, 65].

### MSCs, Neural Stem Cells, and Other Cell Types

Most MSC-based therapies are performed by injecting cells directly into damaged tissues as regenerative therapies [47]. Neural stem cells have also been used to treat brain trauma [83, 84]. Cell implantations can be monitored by most *ex vivo* labeling methods, but to track the month-long cell engraftment process, reporter gene imaging methods that visualize only surviving cells should be considered. For cell tracking for 1–2-week period, ^89^Zr-oxine is applicable to virtually any cell type, but it is imperative to confirm maintenance of cellular functions after labeling.

## Clinical Translation Perspective

^19^F-MRI has been used in clinical trials within the limited field of view. Evaluation of whole-body cell migration is challenging with this method. ^19^F-MRI may better be applied to certain types of cell-based therapies that target specific tissues such as lymph nodes, tumors, or damaged tissues (e.g., regenerative cell therapy). The low sensitivity and requirement of specific MR coils for acquisition for detection of ^19^F are also drawbacks for the wide distribution of ^19^F-MRI. Attempts to increase cell incorporation of ^19^F using cell-penetrating TAT peptide have been successful, and as TAT are in clinical trials for other purposes, this strategy seems promising for advancement of ^19^F-MRI [87].

Whole-body imaging is more straightforward with nuclear medicine methods. ^89^Zr-oxine *ex vivo* cell labeling technology has been gaining popularity since it was first developed in 2015. Efforts have been made to translate ^89^Zr-oxine to the clinic. The generation of GMP-compatible ^89^Zr-oxine has been reported by different groups using different synthesis methods [60, 97], including quality control tests for human use, such as the filter membrane integrity test, endotoxin test, and sterility test [97]. Standardization of GMP-quality ^89^Zr-oxine production will enhance the clinical use of ^89^Zr-oxine.

Radio exposure to organs and whole body in humans in ^89^Zr-oxine PET is expected to be minimal. The human estimated dosimetry of ^89^Zr-oxine from results with labeled NK cells in a rhesus model confirmed the safety of this method [29]. As activated T cells show similar distribution patterns to the liver and spleen [17, 30], it is expected that dosimetry of ^89^Zr-oxine-labeled activated T cells will be similar to that of NK cells. One concern is that ^89^Zr could be released from dying/dead cells and be taken up in bones. This could be problematic when assessing the cell migration to the bone marrow or tissues close to the bone. A solution to this is to add continuous infusion of deferoxamine alone with the labeled cells. The deferoxamine chelates free ^89^Zr, which is then rapidly excreted in the kidneys. Deferoxamine is FDA-approved for the treatment of iron overload and is, therefore, not difficult to add to the regimen of cell infusion.

As cell-based therapies become a major part of cancer treatment, preparation of cell products by commercial third parties has already occurred for DCs and CAR-T cells. With increased production of GMP-quality ^19^F-PFCs and ^89^Zr-oxine, cell labeling with these reagents could also be performed by third parties, making the cell-tracking imaging more routine and less disruptive to workflow, while making multi-site clinical trials possible.

The popularity of genetic engineering of therapeutic cells has lowered the barriers for reporter gene imaging in cell-based therapies. An additional transduction of a reporter gene to the transduction of TCR or CAR in T cells, for example, would be easier to incorporate and justify than attempting to do so only for diagnostic purposes in an otherwise unmanipulated cell. Availability of many clinically used PET tracers compatible with reporter gene imaging [36–38] facilitates the integration of reporter gene PET to evaluate cell-based immunotherapies.

## Concluding Remarks

Direct *ex vivo* cell labeling to track cells in whole body by PET or MRI complements existing therapeutic cell production. Since cell-based immunotherapies usually require cells to be extracted and expanded *ex vivo*, aliquots of the treatment batch can be sequestered for labeling and subsequent imaging. ^89^Zr-oxine PET and ^19^F-PFC MRI technologies hold promise for clinical advancement. Reporter gene PET imaging requires transduction of the cells *ex vivo* but enables long-term tracking of genetically engineered cells. These methods allow quantification of cell distribution after transfer, providing crucial information on the biology of these cells, and act as a tool for monitoring cellular engineering aimed at increasing the homing of the cells to the target tissue or organs. Moreover, pharmacodynamic effects of therapies could be visualized. Selecting an imaging method that best suits the questions being asked is critical for successful cell tracking. Using optimal labeling doses that do not alter viability, phenotype, or functionality of the cells is critical, especially in therapies. Standardization of *ex vivo* cell labeling procedures will make imaging of cell-based immunotherapy more accessible and enhance multi-site clinical trials.
